# Differential activation of G protein‐mediated signaling by synthetic cannabinoid receptor agonists

**DOI:** 10.1002/prp2.566

**Published:** 2020-02-26

**Authors:** Shivani Sachdev, Samuel D. Banister, Marina Santiago, Chris Bladen, Michael Kassiou, Mark Connor

**Affiliations:** ^1^ Department of Biomedical Sciences Macquarie University Sydney NSW Australia; ^2^ The Lambert Initiative for Cannabinoid Therapeutics, Brain and Mind Centre The University of Sydney Sydney NSW Australia; ^3^ School of Chemistry The University of Sydney Sydney NSW Australia

**Keywords:** cannabinoid receptor, G protein, signaling, synthetic cannabinoid receptor agonist, toxicity

## Abstract

Synthetic cannabinoid receptor agonists (SCRAs) are new psychoactive substances associated with acute intoxication and even death. However, the molecular mechanisms through which SCRAs may exert their toxic effects remain unclear—including the potential differential activation of G protein subtypes by cannabinoid receptor type 1 (CB1), a major target of SCRA. We measured CB1‐mediated activation of Gα_s_ and Gα_i/o_ proteins by SCRAs by examining stimulation (pertussis toxin, PTX treated) as well as inhibition (non‐PTX treated) of forskolin (FSK)‐induced cyclic adenosine monophosphate (cAMP) accumulation in human embryonic kidney (HEK) cells stably expressing CB1. Real‐time measurements of stimulation and inhibition of cAMP levels were made using a BRET biosensor. We found that the maximum concentration of SCRAs tested (10 µmol L^−1^), increased cAMP levels 12%‐45% above that produced by FSK alone, while the phytocannabinoid THC did not significantly alter cAMP levels in PTX‐treated HEK‐CB1 cells. All SCRAs had greater potency to inhibit FSK‐induced cAMP levels than to stimulate cAMP levels. The rank order of potencies for SCRA stimulation of cAMP (Gα_s_) was PB‐22 > 5F‐MDMB‐PICA > JWH‐018 ≈ AB‐FUBINACA > XLR‐11. By contrast, the potency of SCRAs for inhibition of cAMP (Gα_i/o_) was 5F‐MDMB‐PICA > AB‐FUBINACA > PB‐22 > JWH‐018 > XLR‐11. The different rank order of potency and E_Max_ of the SCRAs to stimulate Gα_s_‐like signaling compared to Gα_i/o_ signaling suggests differences in G protein preference between SCRAs. Understanding the apparent differences among these drugs may contribute to unravelling their complex effects in humans.

**Abbreviations**
2‐AG: 2‐arachidonoyl glycerol
AC: adenylyl cyclase
BRET: bioluminescence resonance energy transfer
CAMYEL: cAMP sensor YFP‐Epac‐Rluc
CB1: cannabinoid receptor type 1
GIRK: G protein‐gated inwardly rectifying K^+^ channel
HA: hemagglutinin
HEK‐CB1: human embryonic kidney cells stably transfected with HA tagged human CB1 receptors
NPS: novel psychoactive substances
PEI: polyethylenimine
PTX: pertussis toxin
SCRA: synthetic cannabinoid receptor agonists
THC: Δ^9^‐tetrahydrocannabinol

## 1 INTRODUCTION

The use of synthetic cannabinoid receptor agonist (SCRA) new psychoactive substances (NPS) is associated with significant morbidity and mortality compared to use of ∆^9^‐tetrahydrocannabinol (THC), the main psychoactive ingredient of cannabis.^1^, ^2^ SCRAs are linked to a wide range of toxic effects including seizures, agitation, hypertension, cardiotoxicity, kidney damage, and sometimes death.^3^, ^4^ There has been a rapid increase in the number of structurally diverse SCRAs since 2010, with little known about their pharmacology and toxicology at time of identification.^5^ The constant evolution of SCRA structures occurs in response to legislative restriction and development of urine drug screens for existing compounds.^6^, ^7^, ^8^ A time‐series of seizures (by tonnage) of NPS reported to the United Nations Office on Drug and Crime^9^ showed that the SCRAs dominated the synthetic NPS market over the period 2011‐2017.

SCRAs are usually agonists at both cannabinoid type‐1 and type‐2 receptors (CB1 and CB2, respectively^10^); with the psychoactive effects attributed to the activation of CB1.^11^ We have previously described the in vitro quantitative measurement of SCRA efficacy at CB1, where all SCRAs tested showed between 20‐ and 300‐fold greater agonist activity at CB1 compared to THC.^12^ Cannabinoid receptor‐mediated G protein signaling is predominantly through the Gα_i/o_ protein family^13^; however, under some circumstances, CB1 can also stimulate adenylyl cyclase (AC) through Gα_s_‐proteins.^14^, ^15^, ^16^ For example, blockade of the canonical CB1‐Gα_i_ pathway with pertussis toxin (PTX) or sequestration of CB1‐Gα_i_ protein in the primary striatal rat neurons on coexpression with D2 results in an augmentation of cyclic adenosine monophosphate (cAMP) levels by cannabinoids, suggesting that CB1 couples to Gα_s_.^14^, ^15^ A recent study characterized the relationship between CB1 receptor expression and signaling, and showed that at very high receptor expression levels, the effect of CB1 activation on cAMP signaling was stimulatory, a phenotype that was reversed by systematic pharmacological knockdown at the receptor level.^17^ The idea that certain SCRAs may preferentially activate different CB1 Gα subtypes is not unprecedented^18^, ^19^, ^20^; in a study by Costain et al^21^ AB‐CHMINACA elicited an elevation in cAMP levels in both the absence and presence of forskolin (FSK) in human embryonic kidney (HEK) cells transiently expressing CB1, suggesting an AB‐CHMINACA‐specific CB1‐mediated activation of Gα_s_ signaling.

The mechanism(s) through which SCRAs exert different behavioral and physiological effects remains unclear, and which pathways modulated by CB1 activation mediate the specific pharmacological effects of SCRAs is also unknown. Similarly, the question of whether these pathways are activated in a quantitatively or qualitatively similar way by SCRAs and THC is only beginning to be addressed.^22^ Finally, the question of whether SCRA activity at noncannabinoid receptors is also important for their pharmacological effects is very much open.^23^, ^24^, ^25^ With more than 250 SCRAs identified in the NPS market,^9^ elucidation of the differential molecular mechanisms by which these compounds can exert distinct pharmacology, including their signaling via CB1, is essential for understanding their adverse effects. This study examined whether SCRAs that are representative of structural classes confirmed in patients admitted to emergency departments with presumed SCRA toxicity stimulate Gα_s_‐like cAMP signaling via CB1. We measured the SCRA‐mediated stimulation as well as inhibition of FSK‐induced cAMP accumulation in HEK cells stably expressing CB1. We have observed SCRA‐specific CB1‐dependent activation of the two signaling pathways, but THC only coupled to inhibition, not stimulation of cAMP. While AB‐CHIMINACA, previously identified as having a unique profile among SCRAs for elevating cAMP, appeared to signal, in part, through non‐CB1 mechanisms.

## 2 MATERIALS AND METHODS

### 2.1 CB1 receptor transfection and cell culture

HEK 293 FlpIn cells with homogeneous G protein‐gated inwardly rectifying K^+^ (GIRK4) channel expression (the construction of these cells by Grimsey et al will be described elsewhere) were cotransfected with pcDNA5/FRT construct encoding hemagglutinin (HA)‐tagged human CB1 receptor cDNA and pOG44 (Flp recombinase plasmid) using the same random incorporation method of stable transfection as described previously for AtT‐20 pituitary tumor cells.^26^ Cells stably expressing the CB1 receptor were cultured in Dulbecco's Modified Eagle Media (Thermo Fischer Scientific) supplemented with 10% fetal bovine serum (FBS; Sigma‐Aldrich), 100 units mL^−1^ penicillin, 100 µg mL^−1^ streptomycin (Thermo Fischer Scientific), 400 µg mL^−1^ G418 (GIRK4 selection antibiotic) and 100 µg mL^−1^ hygromycin (CB1 selection antibiotic) up to passage 5 (selection phase). Hygromycin concentration was reduced to 80 µg mL^−1^ beyond passage 5 (maintenance phase). Cells were grown in 75 cm^2^ flask at 37°C/5% CO_2_ and passaged at 80% confluency as required. Assays were carried out on cells up to 25 passages.

### 2.2 Assay for cAMP measurement

Intracellular cAMP levels were measured using pcDNA3L‐His‐CAMYEL plasmid, which encodes the cAMP sensor YFP‐Epac‐RLuc (CAMYEL) as outlined in Ref. [27, 28] Cells were detached from the flask using trypsin/EDTA (Sigma‐Aldrich), and resuspended in DMEM supplemented with 10% FBS, 100 units mL^−1^ penicillin, and 100 µg mL^−1^ streptomycin. Cells were seeded in 10 cm dishes at a density of 7 000 000 such that they would be 60%‐70% confluent the next day. On the following day, the cells were transiently transfected with 5 µg of pcDNA3L‐His‐CAMYEL plasmid using the linear polyethylenimine (PEI, m.w. 25 kDa) (Polysciences). The PEI/DNA complex mixture was sequentially added to the cells at the ratio of 1:6, and cells were incubated in 5% CO_2_ at 37°C. Approximately 24 hours after transfection, the cells were then detached from the dish and the pellet was resuspended in Leibovitz's (L‐15—Thermo Fischer Scientific) media supplemented with 1% FBS, 100 units mL^−1^ penicillin, 100 µg mL^−1^ streptomycin and 15 mmol L^−1^ glucose. In the experiments with PTX to irreversibly uncouple Gα_i_ proteins, the cells were resuspended in the media containing 200 ng mL^−1^ PTX. The PTX‐treated and control (non‐PTX treated) cells were plated at a density of 100 000 cells per well in poly D‐lysine (Sigma‐Aldrich) coated, white wall, clear bottomed 96‐well microplates. Cells were incubated overnight at 37°C in ambient CO_2_.

**Figure 1:**
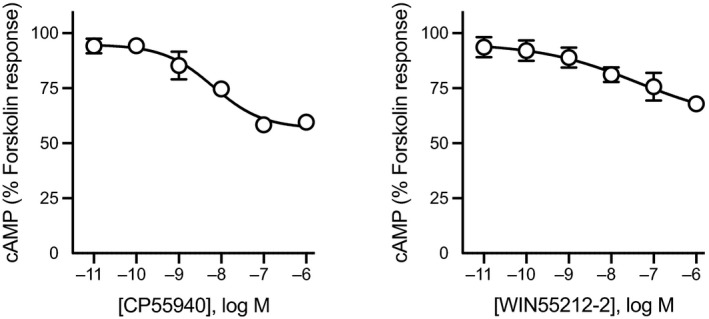
Concentration response curve for CP55940 and WIN55212‐2. Treatment with CP55940 or WIN55212‐2 produced a concentration‐dependent inhibition of forskolin‐mediated cAMP production in human embryonic kidney 293‐cannabinoid receptor type 1. Curves were generated by area under the curve analysis for CP55940 or WIN55212‐2 in the presence of 3 μmol L^−1^ forskolin. Data were normalized to forskolin (100%) and vehicle (0%), and plotted as mean ± SEM for at least five independent experiments performed in duplicate. cAMP, cyclic adenosine monophosphate

The day after plating, FSK (an activator of AC) was prepared in Hanks’ balanced salt solution (HBSS) composed of (mmol L^−1^) NaCl 145, HEPES 22, Na_2_HPO_4_ 0.338, NaHCO_3_ 4.17, KH_2_PO_4_ 0.441, MgSO_4_ 0.407, MgCl_2_ 0.493, CaCl_2_ 1.26, glucose 5.56 (pH 7.4, osmolarity 315 ± 15), and supplemented with 0.1% bovine serum albumin. All the drugs used for the series of real‐time measurements of stimulation and inhibition of cAMP levels were made in 3 µmol L^−1^ of FSK immediately before the assay. The concentration of DMSO (0.10%‐0.13%) was kept constant for all experiments, however this limited the maximum drug concentration that could be tested. Coelenterazine H substrate (NanoLight Technologies) was made in HBSS, and added to a final concentration of 5 µmol L^−1^ (10 µL per well) to the cells, and incubated for 5 minutes after which 10 µL of (10×) drug was added to each well to obtain the desired concentration. A vehicle (HBSS plus DMSO alone) was included in each column of a 96‐well microplate and routinely subtracted from the measurements. The PTX‐treated and control cells were compared side by side. Luminescence was measured using a PHERAstar plate reader (BMG Labtech) at 37°C. The cell signaling was measured at an emission wavelength of 475 and 535 nm simultaneously, and the readings were made every 40 seconds for approximately 20 minutes. A concentration response curve (CRC) for CP55940 and WIN55212‐2 inhibition of cAMP accumulation was performed for each experimental replicate as a reference standard (Figure 1). Day to day variation in the degree of Gs‐stimulation was observed, presumably arising in part from the transient transfections and subsequent PTX treatment required for each assay.

### 2.3 Data analysis

Raw data are presented as inverse bioluminescence resonance energy transfer (BRET) ratio of emission at 475/535 nm, such that an increase in ratio corresponds with increase in cAMP production. Real‐time (raw) cAMP time course data were then analyzed using area under curve analysis in GraphPad PRISM (Graph Pad Software Inc). Data were normalized to the change produced by FSK over 20 minutes (set as 100%) for each experiment. The percent change values were fit to three or four‐parameter non‐linear regression curves in PRISM to derive EC_50_ and *E*_max_. In the three parameter fit the Hill slope was constrained to 1, in the four parameter fit it was free to vary. All final datasets passed the Shapiro‐Wilk test for normality. Unless otherwise stated, the data represent mean ± SEM of at least five independent experiments, each conducted in duplicate. The differences between groups were tested using unpaired Student's *t* test, and one‐way ANOVA as appropriate when comparing multiple groups (PRISM). Statistical significance is defined as *P* < .05.

### 2.4 Materials

CP55940, WIN55212‐2, 2‐arachidonoylglycerol (2‐AG), CUMYL‐4CN‐BINACA, and SR141716A were purchased from Cayman Chemical, THC was from THC Pharm GmbH and was a kind gift from the Lambert Initiative for Cannabis Therapeutics (University of Sydney). PTX was from HelloBio, and FSK was from Ascent Scientific Ltd. All the SCRAs, unless otherwise stated, were synthesized by Dr Samuel D. Banister in the lab of Professor Michael Kassiou at Sydney University. Chemical structure of SCRAs can be found elsewhere.^12^ All the SCRAs were prepared in DMSO and stored in aliquots of 30 mmol L^−1^ in −30°C until needed.

## 3 RESULTS

### 3.1 Real‐time cAMP BRET measurement of the Gα_s_‐mediated signaling of SCRAs

**Figure 2:**
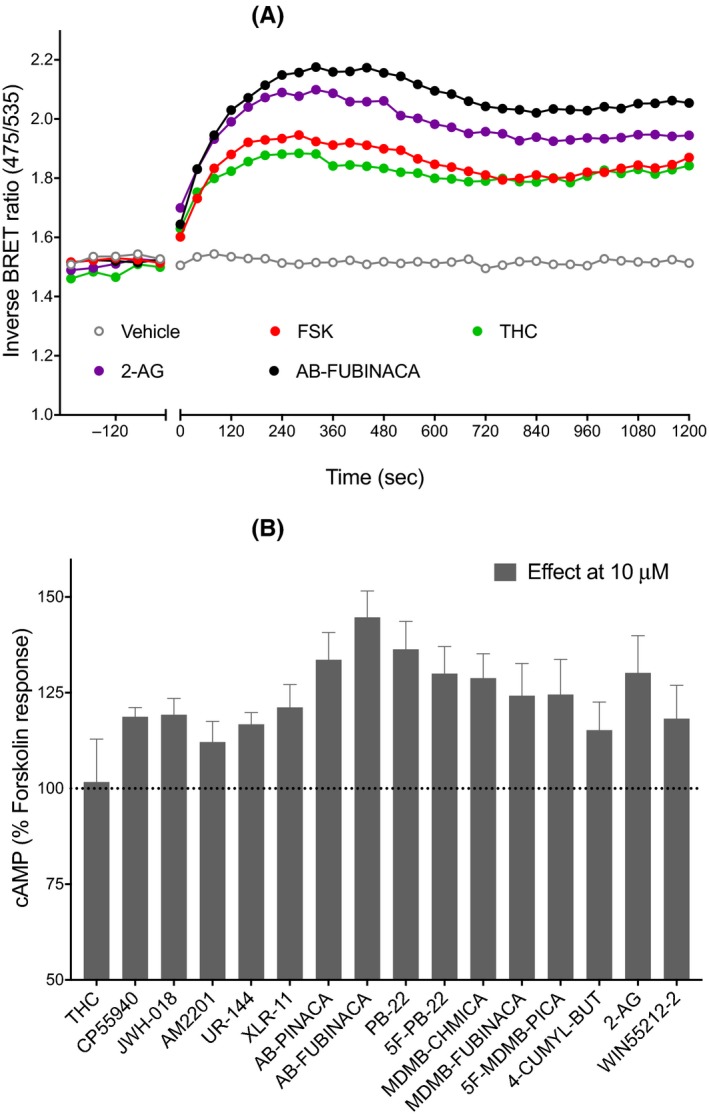
Gα_s_‐mediated signaling of synthetic cannabinoid receptor agonists. A, Representative data for real‐time measurement of stimulation of cAMP levels by 10 μmol L^−1^ of cannabinoids (THC, 2‐arachidinoylglycerol, and AB‐FUBINACA) in human embryonic kidney cells expressing cannabinoid receptor type 1 receptors, an increase in inverse BRET ratio (emission at 475/535 nm) corresponds to an increase in cAMP. B, A bar chart summarizing the cAMP signaling peaks for 16 cannabinoids (excluding AB‐CHMINACA) showing an increase in cAMP levels above that of FSK (3 μmol L^−1^) alone (FSK, 100%). Graphs show mean + SEM for at least five independent experiments performed in duplicate. BRET, bioluminescence resonance energy transfer; cAMP, cyclic adenosine monophosphate; FSK, forskolin; THC, Δ9‐tetrahydrocannabinol

Using the CAMYEL assay, we measured the effect of seventeen cannabinoids (10 µmol L^−1^ each) on the FSK‐stimulated cellular cAMP levels in HEK‐CB1 cells following pretreatment with PTX. All the SCRAs produced an increase in cAMP levels above that produced by FSK alone (100%). Examples of raw traces are shown for some SCRAs (Figure 2A), note that the stimulation of cAMP by SCRAs in the presence of FSK and PTX plateaued approximately after 12 minutes, and maintained at that level for the entire course of the assay (20 minutes). The effects of SCRAs tested ranged from 12% to 45% increase in signal relative to FSK alone. Most of the SCRAs had approximately 1.5 times higher effect than CP55940 (19%) or WIN55212‐2 (18%), except for JWH‐018, UR‐144, AM‐2201, and CUMYL‐4CN‐BINACA, which showed similar or lower effect (Figure 2B). AB‐FUBINACA had up to 2.5 times higher effect than CP55940. In PTX‐treated cells, the endocannabinoid 2‐AG (10 µmol L^−1^) produced an increase in FSK‐stimulated cAMP levels approximately twice that of CP55940, while the phytocannabinoid THC did not significantly alter cAMP levels in the presence of FSK (compared to FSK alone Figure 2B, *P* > .05).

### 3.2 Differential SCRAs‐induced stimulation and inhibition of cAMP signaling in HEK‐CB1

**Figure 3:**
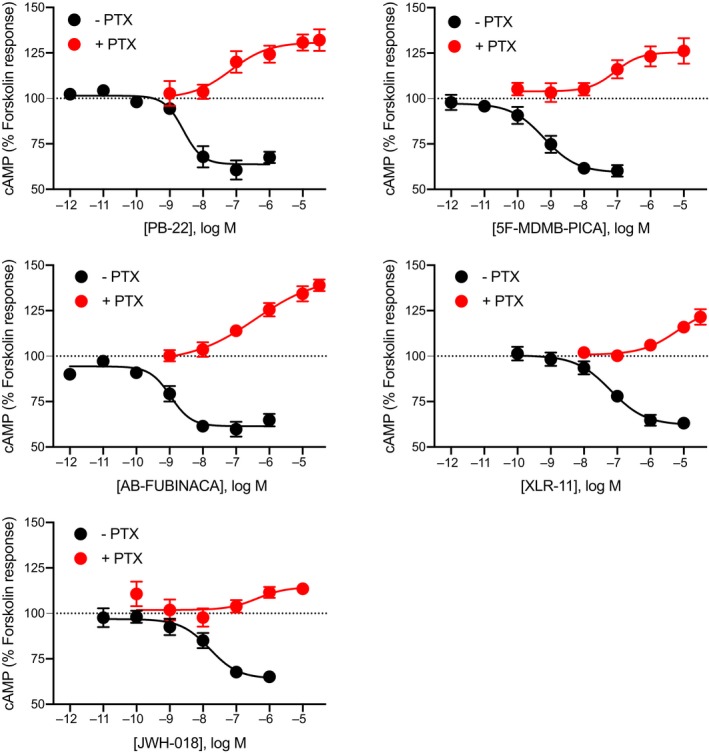
Concentration response curves for SCRAs‐induced stimulation and inhibition of cAMP signaling. Pooled concentration response relationship for five SCRAs (PB‐22, 5F‐MDMB‐PICA, AB‐FUBINACA, XLR‐11, and JWH‐018) for two signaling outputs of cannabinoid receptor type 1—stimulation and inhibition of cAMP levels following overnight treatment in the absence (−PTX, black), or presence (+PTX, red) of PTX. Data were normalized to forskolin (FSK, 100%) and vehicle (0%), and plotted as mean ± SEM for at least five independent experiments performed in duplicate. For some points, the error bars are shorter than the height of the symbol. BRET, bioluminescence resonance energy transfer; cAMP, cyclic adenosine monophosphate; PTX, pertussis toxin; SCRA, synthetic cannabinoid receptor agonist

To assess whether there was any evidence of preferential coupling to Gα_i/o_ over Gα_s_ among SCRAs, we assessed the pharmacological activity (EC_50_ and *E*_max_) of a selection of SCRAs belonging to different structural classes (JWH‐018, PB‐22, AB‐FUBINACA, XLR‐11, and 5F‐MDMB‐PICA), to stimulate and inhibit cAMP in HEK‐CB1 cells. All the SCRAs tested activated CB1 through Gα_i/o_ (inhibitory, non‐PTX treated), and Gα_s_ (stimulatory, PTX treated) in a concentration‐dependent manner (Figure 3). As previously reported,^29^ treatment with CP55940 and WIN55212‐2 produced an immediate concentration‐dependent inhibition of FSK‐mediated cAMP production (*p*EC_50_ CP55940 8.1 ± 0.4, *p*EC_50_ WIN55212‐2 7.9 ± 0.4). All SCRAs had greater potency (0.62‐63 nmol L^−1^) for inhibition of FSK‐induced cAMP levels in non‐PTX‐treated HEK cells compared to their potency to stimulate cAMP levels (69‐4720 nmol L^−1^) (Table 1). The activation of CB1‐Gα_s_ by SCRAs showed a wide variation in *E*_max_ values, and there was a significant difference in efficacy between AB‐FUBINACA, XLR‐11 and JWH‐018 (one‐way ANOVA, *P* < .05). The rank order of efficacy for stimulation of Gαs was AB‐FUBINACA ≈ PB‐22 > 5F‐MDMB‐PICA > XLR‐11 > JWH‐018, whereas all the SCRAs were similarly effective at inhibiting cAMP production (Table 1). It should be noted that the CRC for the most efficacious compound tested at Gα_s_ pathway, AB‐FUBINACA, may not have reached a plateau at highest concentration we could test, 30 µmol L^−1^, and that of XLR‐11 almost certainly had not. The first SCRA to be identified in spice, JWH‐018, caused partial (14% increase over FSK alone) activation of Gα_s_ pathway, but produced greater inhibition of the FSK‐induced cAMP response (64% of FSK response). Whereas other SCRAs tested in this study induce moderate activation of Gα_s_ pathway (26%‐36% relative to FSK) compared to their activity at Gα_i/o_ inhibitory pathway (Figure 3). The rank order of potencies for SCRAs for inhibition of cAMP (Gα_i/o_) is 5F‐MDMB‐PICA > AB‐FUBINACA > PB‐22 > JWH‐018 > XLR‐11. By contrast, the potency of SCRAs for stimulation of cAMP (Gα_s_) is PB‐22 > 5F‐MDMB‐PICA > JWH‐018 ≈ ≥ AB‐FUBINACA > XLR‐11. The most efficacious SCRA at Gα_s_ pathway (AB‐FUBINACA) was roughly 300 times less potent at Gα_s_ than the Gα_i/o_‐pathway, while JWH‐018 was only 18 times less potent. XLR‐11 had much lower potency compared to all the other SCRAs for both Gα_s_ pathway and Gα_i/o_ pathway (Table 1).

**Table 1:** Comparison of pharmacological activity (EC_50_ and *E*_max_) of SCRAs‐induced stimulation (*G*_s _(+PTX)) and inhibition (*G*_i _(−PTX)) of cAMP signaling in HEK‐CB1 cells

Compound	*G*_i _(‐PTX)		*G*_s _(+PTX)		*G*_i_ (−PTX) selectivity
	*p*EC_50_ (EC_50_, nmol L^−1^)	*E*_max_ (% FSK)	*p*EC_50_ (EC_50_, nmol L^−1^	*E*_max_ (% FSK)	
CP55940	8.1 ± 0.4 (7)	58 ± 3	—	—	—
WIN55212‐2	7.9 ± 0.4 (11)	70 ± 4	—	—	—
JWH‐018	7.8 ± 0.2 (16)	64 ± 3	6.5 ± 0.7 (288)	114 ± 4	18
XLR‐11	7.2 ± 0.2 (63)	63 ± 2	5.3 ± 0.8 (4720)	124 ± 5	75
PB‐22	8.6 ± 0.2 (2.5)	64 ± 3	7.2 ± 0.5 (69)	130 ± 3	28
AB‐FUBINACA	9.0 ± 0.2 (0.96)	61 ± 2	6.4 ± 0.5 (278)	144 ± 12	290
5F‐MDMB‐PICA	9.2 ± 0.2 (0.62)	60 ± 4	7.1 ± 0.4 (85)	126 ± 5	137

Note

The selectivity is expressed as the ratio of *G*_s _(+PTX) EC_50_ to *G*_i _(−PTX) EC_50_. Pooled data from at least five independent experiments was fit to a three parameter logistic equation in PRISM. Data are presented ± SEM.

Abbreviations: cAMP, cyclic adenosine monophosphate; CB1, cannabinoid receptor type 1; FSK, forskolin; HEK, human embryonic kidney; PTX, pertussis toxin; SCRA, synthetic cannabinoid receptor agonist.

**Figure 4:**
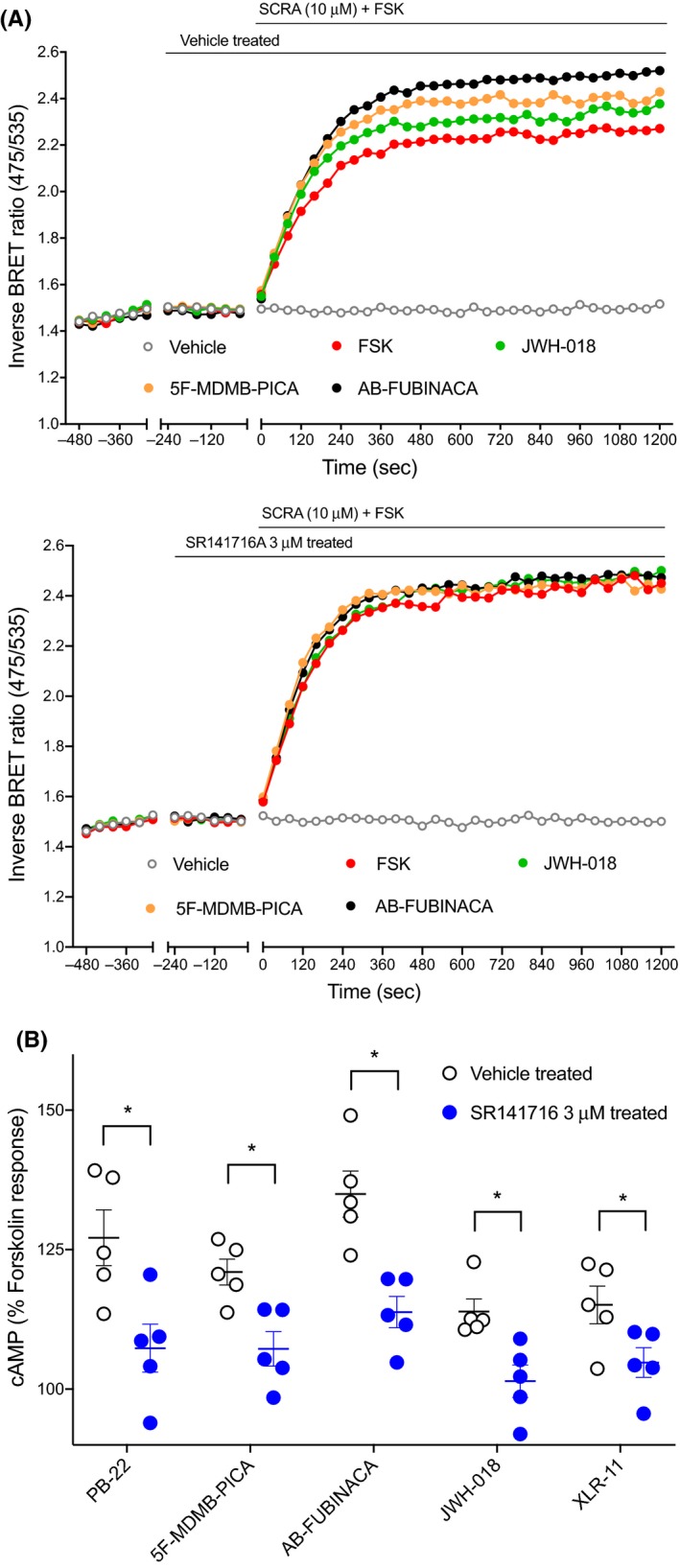
Effect of CB1 antagonist on the SCRA‐mediated cAMP signaling peaks in HEK‐CB1 cells. A, Traces from a representative experiment showing that SCRA (JWH‐018, 5F‐MDMB‐PICA, and AB‐FUBINACA) induced observed stimulatory effects were inhibited by SR141716A (CB1 antagonist, 3 μmol L^−1^) pretreatment. B, Scatter dot plot representing SCRAs‐mediated stimulation of forskolin (3 µmol L^−1^)‐induced cAMP response in presence and absence of SR141716A 3 µmol L^−1^ on HEK 293 cells expressing CB1. Within each set SCRAs (10 µmol L^−1^) were compared to SCRAs + SR141716 (unpaired Student's t test, *P* < .05 marked with *). Data were normalized to forskolin (FSK, 100%) and vehicle (0%), and plotted as mean ± SEM for at least five independent experiments performed in duplicate. cAMP, cyclic adenosine monophosphate; CB1, cannabinoid receptor type 1; HEK, human embryonic kidney; SCRA, synthetic cannabinoid receptor agonist

We then tested if the SCRA‐induced observed stimulatory effects were mediated through CB1 receptors. Pretreatment of HEK‐CB1 with SR141716A (3 μmol L^−1^, 5 minutes), a potent and selective CB1 antagonist,^30^ prevented the subsequent SCRA (10 µmol L^−1^)‐mediated stimulation of FSK‐induced cAMP response compared to the vehicle‐treated cells (Figure 4; *P* < .05). Consistent with Gα_s_ CB1‐specific responses of SCRAs, pretreatment with SR141716A also blocked the inhibitory cAMP signaling induced by SCRAs (Figure S1; *P* < .05).

**Figure 5:**
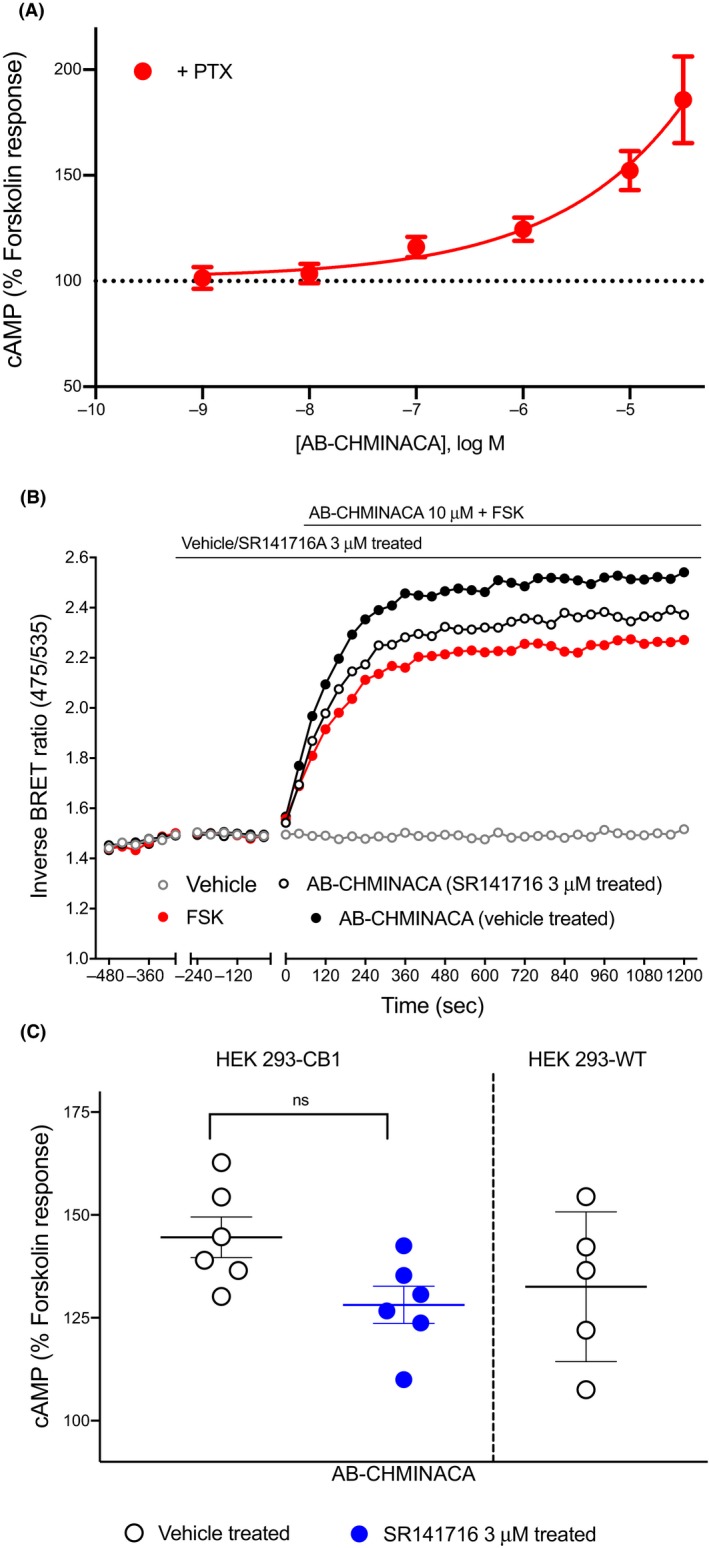
AB‐CHMINACA does not modulate cAMP levels via CB1 receptors in HEK 293 cells. A, Treatment with AB‐CHMINACA produced a concentration‐dependent increase in forskolin‐mediated cAMP production in HEK 293‐CB1 in presence of PTX. B, Traces from a representative experiment showing that AB‐CHMINACA (10 µmol L^−1^) induced observed stimulatory effects were only partially inhibited by SR141716A 3 µmol L^−1^. C, Scatter dot plot comparing AB‐CHMINACA‐mediated stimulation of forskolin (3 μmol L^−1^)‐induced cAMP response in presence and absence of SR141716 3 µmol L^−1^ in HEK 293‐CB1 cells, and the data were not significantly different. AB‐CHMINACA (10 µmol L^−1^) also modestly augmented forskolin‐stimulated cAMP levels in HEK‐wild‐type cells (not containing CB1 receptors). Graphs show mean ± SEM for at least five independent experiments performed in duplicate. cAMP, cyclic adenosine monophosphate; CB1, cannabinoid receptor type 1; HEK, human embryonic kidney; PTX, pertussis toxin

AB‐CHMINACA has previously been reported to stimulate Gα_s_‐like cAMP signaling pathway in a concentration‐dependent manner in HEK‐CB1 cells.^21^ Following PTX treatment, AB‐CHMINACA increased cAMP levels above that of FSK alone (Figure 5A) in a concentration‐dependent manner, with an increase of 86 ± 21% at 30 µmol L^−1^. However, in cells pretreated with SR141716A (3 μmol L^−1^, 5 minutes), the stimulatory effects of AB‐CHMINACA (10 µmol L^−1^) were only partially inhibited, in contrast to other SCRAs tested in this study (Figure 5B). To confirm that this response was at least in part non‐CB1‐mediated, AB‐CHMINACA was tested in HEK 293 wild‐type cells; in these cells, AB‐CHMINACA (10 µmol L^−1^) also produced a small increase in FSK‐stimulated cAMP accumulation (Figure 5C, 29 ± 10%), suggesting that some of these stimulatory effects were occurring via mechanism(s) unrelated to CB1 receptor activity.

## 4 DISCUSSION

In this study, we set out to systematically characterize the ability of several SCRAs to activate Gα_s_ and Gα_i/o_ proteins by examining stimulation as well as inhibition of FSK‐induced cAMP accumulation in HEK cells stably expressing CB1. Assays of cAMP signaling revealed that the maximum concentration of SCRAs tested (10 µmol L^−1^), increased cAMP levels 12%‐45% above that produced by FSK alone, while THC failed to increase cAMP levels, an observation consistent with the findings of Finlay et al^17^ To further investigate the differential response of SCRA‐induced activation and inhibition of cAMP production, we constructed the CRCs for SCRAs belonging to different structural classes (JWH‐018, PB‐22, AB‐FUBINACA, XLR‐11, and 5F‐MDMB‐PICA); the rank order of potency of these SCRAs to stimulate Gα_s‐_like cAMP signaling pathway was different from their activity in Gα_i/o_‐pathway (inhibition of cAMP), suggesting that some of these drugs differentially regulate G protein coupling to CB1.

SCRA‐mediated inhibition of cAMP has been extensively studied in cell models expressing cannabinoid receptors^21^, ^24^ but some studies have also demonstrated the ability of cannabinoids to stimulate Gα_s_‐like cAMP signaling downstream of CB1.^14^, ^15^, ^16^, ^17^ We found that, at a concentration of 10 µmol L^−1^, three of the fifteen SCRAs tested, AB‐FUBINACA, PB‐22, and AB‐PINACA, activated Gα_s_‐like CB1 signaling to more than 30% above the FSK response. In a previous study using AB‐CHMINACA, Costain et al^21^ showed similar increases in cAMP levels to that seen in this study without the need for FSK or PTX pretreatment. In our cells, none of the cannabinoids tested altered cAMP levels in the absence of FSK (data not shown). Costain et al^21^ performed their assays on HEK293T cells transiently transfected with CB1. Transient transfection of CB1 may have led to a higher level of receptor expression than in our cells, and high levels of CB1 receptor expression are sufficient to result in a switch in cAMP signaling from Gα_i_‐mediated (inhibitory) to Gα_s_‐mediated (stimulatory) nett effect.^17^ Costain *et al* 21 also used a GloSensor cAMP assay, wherein cannabinoid was added for 12 minutes prior to the addition of FSK (10 µmol L^−1^), and luminescence was monitored for 30 minutes.^21^ This may have contributed to the differences in the results of the two studies, but it is not immediately obvious why this would be. Finally, the HEK‐293 “T” subclone used in the previous study harbors considerable genomic differences to the parental HEK 293 cell line used in this study,^31^, ^32^ which may also contribute to altered cAMP responses (via different AC isoforms). However, our data, together with that of Costain et al^21^ suggest potentially different receptor/effector coupling pathways in the presence of some SCRAs (AB‐FUBINACA, PB‐22, and AB‐PINACA, AB‐CHMINACA) compared to other CB1 ligands.

We further sought to investigate SCRA differential activation of distinctive G protein subsets—inhibition and stimulation of FSK‐mediated cAMP signaling. The relative ability of SCRAs to induce inhibition of cAMP production via Gα_i/o_ is very similar to that observed in previous studies in assays of membrane potential and [^35^S]GTPγS binding.^12^, ^25^, ^33^, ^34^ The similar *E*_max_ observed for the SCRA‐mediated activation of Gα_i/o_‐CB1 signaling in this study probably reflects receptor reserve for inhibition of cAMP accumulation in these cells, wherein maximal responses are elicited at less than maximal receptor occupancy because the system maximum is already achieved.^12^ SCRA‐induced stimulation of cAMP showed significant differences in *E*_max_ (Table 1), suggesting an absence of receptor reserve for most of the Gs‐dependent signaling we observed for the SCRA in these conditions. This may (at least for the drugs with a lower *E*_max_) reflect an accurate representation of intrinsic efficacy of the ligands at this pathway.^35^ The observed dynamic range of *E*_max_ for cannabinoids is consistent with CB1 having low coupling efficiency to both Gα_s_ pathway and β‐arrestin‐2 (as observed previously^32^), compared to that of Gα_i_ pathway.^17^, ^36^, ^37^ Future studies could examine the structure of SCRA‐bound CB1‐Gα_s_ complexes, which might assist in explaining the observed cAMP signaling profiles. This is particularly interesting given that the interaction of SCRA MDMB‐FUBINACA with the “toggle twin switch” in the CB1 binding pocket coupled to Gα_i_ was recently studied.^38^ The rigid C‐shape geometry of MDMB‐FUBINACA along with the strong pi‐pi interaction of its indazole ring with “toggle twin switch” residues, might help distinguish the high efficacy agonist activity of SCRA from partial agonists like THC lacking “toggle twin switch” interaction.^38^ Promiscuous coupling to both Gα_i_ and Gα_s_ has been reported for multiple GPCRs (eg β_2_‐adrenergic receptor),^39^ while some receptors couple predominantly to one G protein subtype (eg μ‐opioid receptor coupling to the Gα_i/o_ family^40^). The potential of cannabinoids to differentially activate one signaling cascade over another (functional selectivity^41^) may aid the development of new therapeutic compounds with reduced psychoactive effects; a research domain that has attracted much recent interest.^42^

Considering the adverse effects associated with SCRA use, it is important to continue characterizing the pharmacological profile of these compounds in order to understand the mechanisms driving their toxicity.^43^, ^44^ Although this study does not identify which pathway contributes to the toxic effects observed following SCRA consumption, our data do provide valuable insights into SCRA‐mediated stimulation and inhibition of cAMP signaling in vitro. Previous studies have shown that JWH‐018‐ AM‐2201‐, 5F‐AB‐PINACA‐, and CUMYL‐4CN‐BINACA‐induced seizures are CB1‐mediated in mice, which might explain some of the toxicity experienced by recreational users of these drugs.^43^, ^44^, ^45^, ^46^, ^47^, ^48^, ^49^ Our data shows that SCRA‐induced cAMP increase was abolished after SR141716A treatment, supporting the hypothesis that SCRAs Gα_s_‐like effects were mediated through CB1 receptor. All the SCRAs tested in this study exhibited greater potency at Gα_i_‐ than Gα_s_‐like pathways, and the efficacies of these SCRAs have previously been measured in response to Gα_i_‐mediated activation of GIRK channel in AtT20‐CB1 cells.^12^ The rank order of SCRA efficacy based on selectivity for Gα_i_‐GIRK signaling was found to be 5F‐MDMB‐PICA > XLR‐11 > AB‐FUBINACA > PB‐22 ≈ JWH‐018.^12^ 5F‐MDMB‐PICA showed the highest efficacy for modulation of K channel activity via Gα_i_ pathway in the former study, in contrast to the intermediate efficacy of 5F‐MDMB‐PICA to stimulate the Gα_s_‐like cAMP signaling pathway in this study. AB‐FUBINACA exhibited greater efficacy for the Gα_s_ pathway compared to its Gα_i_‐mediated activity profile in the membrane potential assay.^12^ Evaluating the differences in G protein preference between SCRAs may be an important part of understanding the apparent differences in effect between these drugs in humans. However, the biological significance of SCRA‐mediated differential coupling of CB1 to G_i/o_ and G_s_ is not well understood. The G_s_ signaling of CB1 arises in circumstances where G_i/o_ is exhausted or sequestered, and has been measured after PTX treatment or when other G_i_‐coupled receptors are concomitantly activated. The phenomenon was first observed in primary rat striatal neurons natively expressing CB1 and D2 receptors,^14^, ^15^ while a switch in G_i_‐G_s_ signaling due to high CB1 expression has subsequently been defined in recombinant systems.^17^ The phenomenon of CB1‐Gs coupling may be relevant in specific cancer conditions where upregulation in CB1 receptor was reported (eg colorectal cancer, human epithelia ovarian tumors, and prostate cancer).^17^

Our study showed that SCRAs have significantly different pharmacological profiles (maximal activities and potencies) for the activation of CB1‐mediated G protein‐stimulation and ‐inhibition of FSK‐mediated cAMP signaling. Although it is speculated that the adverse effects of SCRAs are mediated by CB1,^49^, ^50^ based on the results presented here we wonder how the differential responses of SCRAs are related to the physiological effects resulting from the activation of each intracellular pathway, and if these may be correlated with the in vivo toxicity of SCRAs. The unique toxicological profile of SCRAs may result from a combination of factors; pharmacokinetic differences, activity at both cannabinoid and noncannabinoid targets, pharmacological activity of metabolites and thermolytic degradants.^25^, ^37^, ^51^, ^52^, ^53^ These findings may provide a starting point to help predict the pharmacological characteristics of SCRAs that demonstrate differential activation of Gα_i_ vs Gα_s_ coupling to CB1.

## DISCLOSURES

The authors declare that they have no conflict of interest related to this work.

## AUTHORS’ CONTRIBUTIONS

SS designed and performed experiments, analyzed the data and wrote the manuscript. SDB synthesized SCRAs supervised by MK. MS and CB advised on the CAMYEL assay and data analysis. MC provided critical feedback and helped shape the research, analysis, and manuscript. All authors reviewed and edited the manuscript.
