# Supplementary material for: Differential activation of G protein‐mediated signaling by synthetic cannabinoid receptor agonists
Source: Pharmacol Res Perspect. 2020 Feb 26;8(2):e00566. doi: 10.1002/prp2.566 (PMC7043210; doi:10.1002/prp2.566)
Supplement: Supplementary file 1 [file PRP2-8-e00566-s001.pdf]

Supplementary Figure 1

Effect of CB1 antagonist on the SCRA induced inhibition of cAMP signalling. A. Traces from a representative experiment showing that SCRA (JWH-018, 5F-MDMB-PICA, and AB-FUBINACA) induced inhibitory effects were completely blocked by SR141716A (CB1 antagonist, 3 μM) pre-treatment. **B**. Scatter dot plot representing SCRAs-mediated inhibition of forskolin-induced cAMP response in presence and absence of SR141716A 3 μM on HEK 293 cells expressing CB1. Within each set SCRAs (100 nM) were compared to SCRAs + SR141716. Data were normalized to forskolin (3 μM) (100%) and vehicle (0%), and plotted as mean ± SEM for at least 5 independent experiments performed in duplicate.

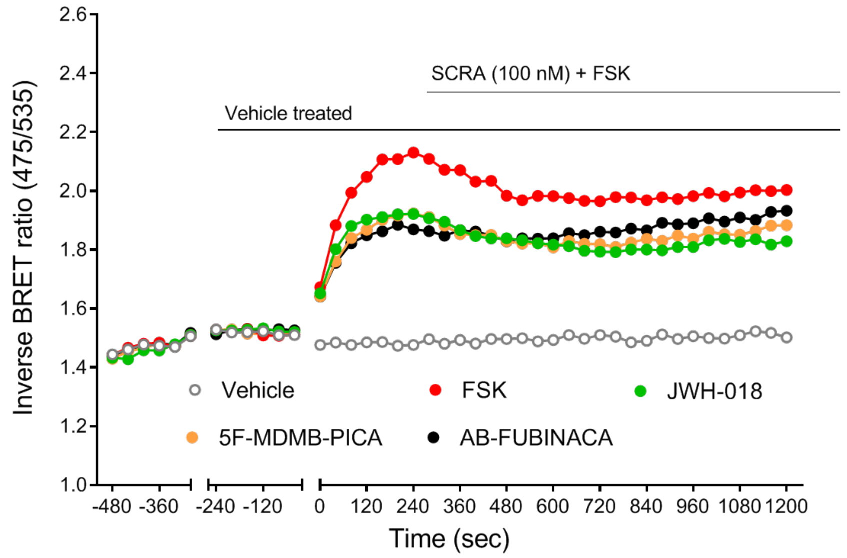

Inverse BRET ratio (475/535)
SCRA (100 nM) + FSK
Vehicle treated
Vehicle
FSK
JWH-018
5F-MDMB-PICA
AB-FUBINACA
Time (sec)

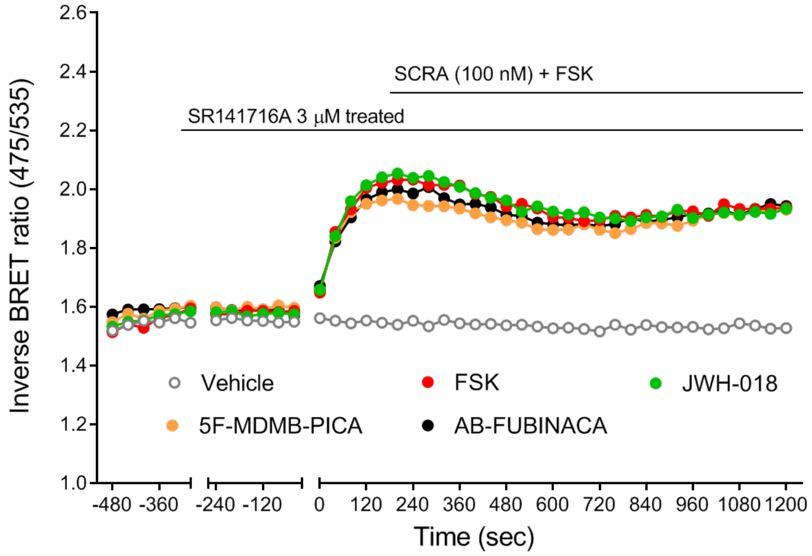

Inverse BRET ratio (475/535)
SCRA (100 nM) + FSK
SR141716A 3 μM treated
Vehicle
FSK
JWH-018
5F-MDMB-PICA
AB-FUBINACA
Time (sec)

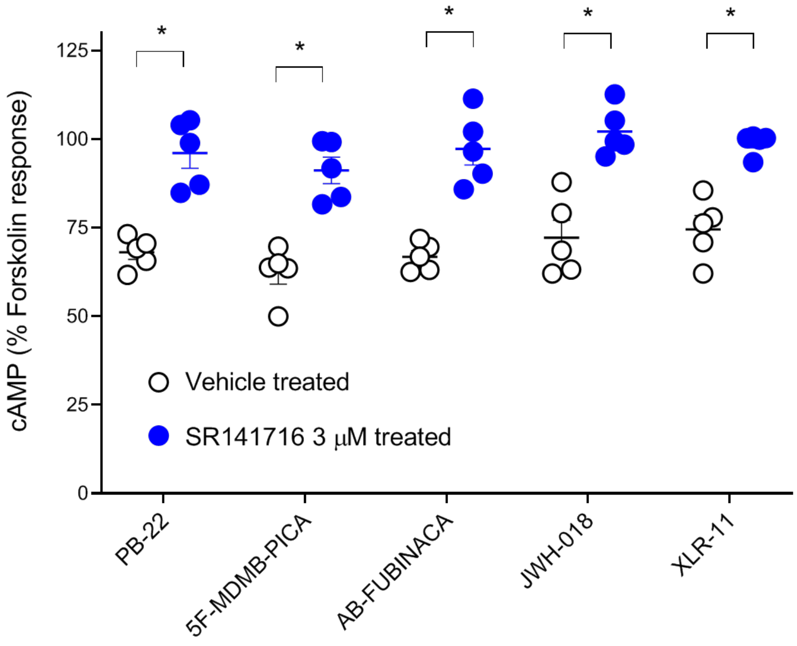

cAMP (% Forskolin response)
Vehicle treated
SR141716 3 μM treated
PB-22
5F-MDMB-PICA
AB-FUBINACA
JWH-018
XLR-11

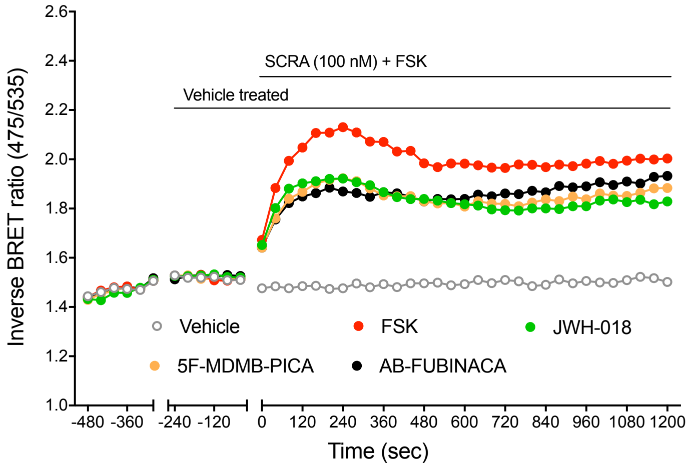
Inverse BRET ratio (475/535)
SCRA (100 nM) + FSK
Vehicle treated
Vehicle
FSK
JWH-018
5F-MDMB-PICA
AB-FUBINACA
Time (sec)

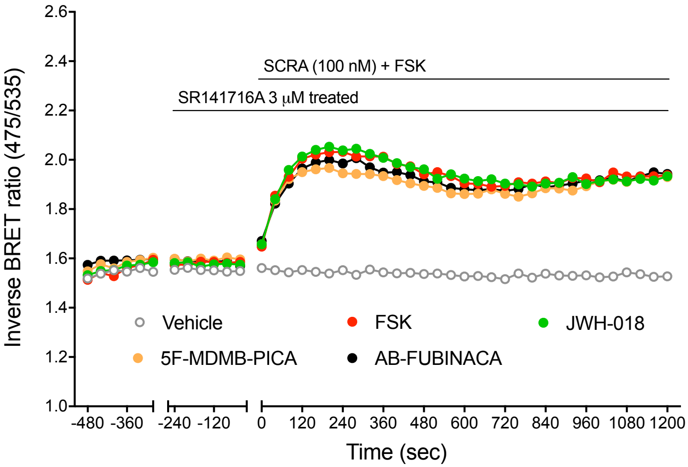
Inverse BRET ratio (475/535)
SCRA (100 nM) + FSK
SR141716A 3 μM treated
Vehicle
FSK
JWH-018
5F-MDMB-PICA
AB-FUBINACA
Time (sec)

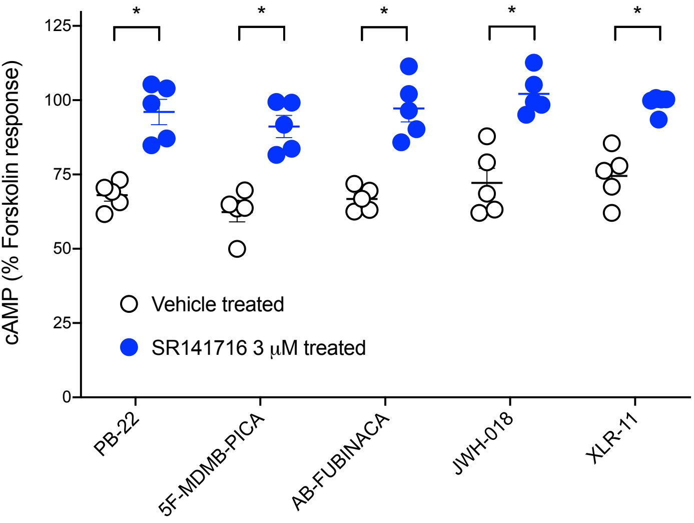
cAMP (% Forskolin response)
Vehicle treated
SR141716 3 μM treated
PB-22
5F-MDMB-PICA
AB-FUBINACA
JWH-018
XLR-11
