# Supplementary material for: Differential activation of G protein‐mediated signaling by synthetic cannabinoid receptor agonists
Source: Pharmacol Res Perspect. 2020 Feb 26;8(2):e00566. doi: 10.1002/prp2.566 (PMC7043210; doi:10.1002/prp2.566)
Supplement: Supplementary file 2 [file PRP2-8-e00566-s002.docx]

**Supplementary Table 1**

Comparison of pharmacological activity (EC_50_ and E_MAX_) of SCRAs-induced stimulation (*G_s_* (+PTX)) and inhibition *(G_i_* (-PTX)) of cAMP signalling in HEK-CB1 cells. Data was fit to a 4 parameter logistic equation in PRISM. The selectivity is expressed as the ratio of *G_s_* (+PTX) EC_50_ to *G_i_* (-PTX) EC_50_. Data is presented ± s.e.

| Compound | *G_i_* (-PTX) | | *G_s_* (+PTX) | | *Gi* (-PTX) selectivity |
| --- | --- | --- | --- | --- | --- |
|  | *p*EC_50_  (EC_50_, nM) | E_max_ (% FSK)  (nH) | pEC_50_  (EC_50_, nM) | E_max_ (% FSK)  (nH) |  |
| CP55940 | 8.2 ± 0.3  (6.4) | 57 ± 5  *-0.7 ± 0.3* | - | - | - |
| WIN55212-2 | 7.4 ± 2  (40) | 60 ± 9  *-0.4 ± 0.5* | - | - | - |
| JWH-018 | 7.8 ± 0.3  (16) | 64 ± 4  *-0.9 ± 0.5* | 6.7 ± 0.7  (221) | 113 ± 4  *1.4 ± 2* | 14 |
| XLR-11 | 7.2 ± 0.2  (63) | 62 ± 3  *-0.8± 0.3* | 5.2 ± 0.8  (6490) | 127 ± 16  *0.8 ± 0.6* | 103 |
| PB-22 | 8.6 ± 0.2  (2.5) | 64 ± 3  *-1.5 ± 0.6* | 7.2 ± 0.5  (69) | 131 ± 5  *0.8± 0.7* | 28 |
| AB-FUBINACA | 9.0 ± 0.2 (1.1) | 61 ± 2  *-1.4 ± 1* | 6.4 ±0.5  (383) | 144 ± 12  *0.5 ± 0.3* | 348 |
| 5F-MDMB-PICA | 9.2 ± 0.2 (0.62) | 59 ± 4  *-0.9 ± 0.4* | 7.1 ± 0.4  (85) | 126 ± 5  *1.1 ± 1.5* | 137 |
